# Comparison of national cross-sectional breast-feeding surveys by maternal education in Europe (2006–2016)

**DOI:** 10.1017/S1368980018002999

**Published:** 2018-12-05

**Authors:** Mahesh Sarki, Alexandr Parlesak, Aileen Robertson

**Affiliations:** 1 Global Health, University of Copenhagen, Copenhagen, Denmark; 2 Global Nutrition and Health, University College Copenhagen, Sigurdsgade 26, 2200 København N, Denmark

**Keywords:** Exclusive breast-feeding, Maternal education, Social gradient, Inequality, Europe

## Abstract

**Objective:**

Breast-feeding is an important determinant of health of mothers and their offspring. The present study aimed to compare breast-feeding rates across Europe disaggregated by maternal education and establish what proportion achieves at least 50 % exclusive breast-feeding (EBF) at 6 months.

**Design/Setting:**

Secondary analysis of national or sub-national studies’ breast-feeding data for EU Member States plus Norway and Iceland, published in 2006–2016. Nineteen EU Member States plus Norway reported rates of EBF and any breast-feeding disaggregated by maternal education, of which only thirteen could be matched to the International Standard Classification of Education.

**Participants:**

Mothers and their infants aged 0–12 months.

**Results:**

Data on EBF rates at 6 and 4 months were found in only four and six countries, respectively. At 6 months, EBF rates of 49 % in Slovakia and 44 % in Hungary were closest to WHO’s target of at least 50 % EBF. At 4 months, mothers with high education level in Denmark, the Netherlands and Germany had the highest EBF rates (71, 52 and 50 %, respectively). Mothers with low education level were less likely to initiate breast-feeding and cessation occurred early. The inequality gap ranged from 63 % in Irish mothers to no gap or very low levels of inequality in Poland, Sweden and Norway.

**Conclusions:**

More mothers with high, compared with low, education initiate breast-feeding and practise EBF for longer. More European policies should be targeted to protect, support and promote breast-feeding, especially among mothers with only mandatory education.

WHO Member States have agreed six Global Targets for Nutrition^(^ ^1^ ^)^. One target is to increase the rate of exclusive breast-feeding (EBF) in the first 6 months to at least 50 % by 2025 and another target is to halt the increase in childhood overweight. Globally, breast-feeding rates have not improved substantially over recent years and rates of EBF at 6 months are well below the 2025 target in most countries. In low- and middle-income countries reliable monitoring systems report breast-feeding rates less than 40 % EBF in the first 6 months^(^ ^2^ ^)^, but the average rates in low- and middle-income countries are high compared with those in high-income countries. Interestingly, it appears in low- and middle-income countries that women with low socio-economic status (SES) breast-feed longer than those with high SES, in contrast to high-income countries where the trend appears to be in the opposite direction^(^ ^3^ ^)^. Mothers of low SES in Europe appear less likely to initiate breast-feeding and cessation occurs early compared with those of high SES^(^ ^4^ ^–^ ^7^ ^)^. Moreover, infants who are predominantly formula-fed compared with those being exclusively breast-fed for the first 6 months are two-and-a-half times more likely to be obese at 24 months^(^ ^8^ ^)^ and cessation of EBF before 4 months increases the risk of childhood obesity^(^ ^9^ ^,^ ^10^ ^)^. Indeed, the prevalence of obesity is also greater among low- compared with high-SES families^(^ ^8^ ^,^ ^11^ ^–^ ^13^ ^)^ and obese mothers especially have to overcome more barriers when trying to breast-feed^(^ ^14^ ^,^ ^15^ ^)^. It has been suggested that an improvement in EBF rates among low-SES mothers could help reduce health inequalities^(^ ^16^ ^,^ ^17^ ^)^ related to obesity^(^ ^16^ ^)^ and resulting morbidity^(^ ^3^ ^,^ ^17^ ^,^ ^18^ ^)^.

Because of the lack of published studies comparing the rates of breast-feeding across socio-economic groups in Europe^(^ ^19^ ^,^ ^20^ ^)^, the second recommendation in the report *Diet, Nutrition and Obesity: Infant Feeding by Socioeconomic Status* stated that:

*‘Data on breastfeeding prevalence and complementary feeding practices by socio-economic status do exist in many EU Member States. … However, apart from one comparative survey published in 2012 by Ibanez et al., no systematic comparison has been made across EU Member States. It is therefore recommended to collect and compare existing data from each EU Member State on breastfeeding prevalence and complementary feeding practices by socio-economic status (i.e. education, profession, maternity protection, etc.).’*

Therefore, the present study aimed to compare breast-feeding rates using the level of maternal education as a proxy for SES^(^ ^21^ ^)^. Given the health benefits for both mothers and their infants^(^ ^2^ ^)^, low breast-feeding rates in low-SES families are likely to result in high rates of health inequalities which could be prevented through appropriate policy actions.

## Methods

A combination of search methods was used between May 2016 and November 2017. This included an Internet-based search of official websites belonging to national authorities along with websites of national and international organizations dealing with breast-feeding. Where national surveys were not found, databases including MEDLINE, CINAHL, EMBASE, Cochrane, PSYCInfo, PubMed and Google Scholar were searched using the keywords: ‘breastfeeding’ AND/OR ‘infant and young child feeding practices’, ‘breastfeeding prevalence’ and ‘breastfeeding statistics’. Data from the most recent breast-feeding survey carried out in each country were sought. The earliest survey was published in 2006 and the most recent in 2016.

To help find surveys published in national languages, Google Translate was used followed by thorough validation of data translations by native speakers knowledgeable about breast-feeding. For example, the national coordinators of the national Baby Friendly Hospital Initiative (Table 1)^(^ ^22^ ^)^ were asked to confirm national breast-feeding prevalence (personal communications, May–October 2016). Failing contact with a national coordinator, native speakers at the department of Global Nutrition and Health at University College Copenhagen were asked to confirm translations in their national languages.

**Table 1:** Levels of maternal education defined according to ISCED classification (low = ISCED 0–2; middle = ISCED 3–4; high = ISCED 5–6)

Country	Indicators used for level of maternal education in national surveys or studies	Classification based on ISCED
Austria^(^ ^26^ ^,^ ^27^ ^)^	Compulsory education	Low
	Apprenticeship	Middle
	Intermediate vocational school	
	Secondary school certificate	
	Secondary school certificate, nursing school	
	University degree	High
Belgium	No data by maternal education	
Bulgaria^*^ ^(^ ^28^ ^,^ ^29^ ^)^	No education	Low
	Primary	
	Elementary	
	Secondary	Middle
Croatia^(^ ^30^ ^,^ ^31^ ^)^	Primary school	Low
	Secondary school	Middle
	Higher education	High
Cyprus	No data by maternal education	
Czech Republic	No data by maternal education	
Denmark^(^ ^32^ ^–^ ^34^ ^)^	Primary school	Low
	Vocational education	Middle
	General or vocational upper secondary education	
	Short or intermediate higher education	High
	Long higher education	
Estonia	No data by maternal education	
Finland^(^ ^35^ ^,^ ^36^ ^)^	Basic	Low
	Upper secondary level	Middle
	Lower university degree or lowest level of tertiary education	High
	Highest university degree or postgraduate education	
France^(^ ^37^ ^,^ ^38^ ^)^	Less than Baccalauréat level	Low
	Baccalauréat level	Middle
	Above Baccalauréat level	High
Germany^(^ ^39^ ^)^	Low	Low
	Medium	Middle
	High	High
Greece^(^ ^40^ ^,^ ^41^ ^)^	No school at all	Low
	Some primary classes	
	Primary school graduate	
	Third high school class	Middle
	High school graduate	
	Graduate technological education institutions (TEI)	High
	University graduate	
Hungary	No data by maternal education	
Ireland^(^ ^42^ ^,^ ^43^ ^)^	Lower secondary or less	Low
	Leaving certificate	Middle
	Sub-degree	
	Degree or third level	High
Italy^(^ ^44^ ^,^ ^45^ ^)^	Compulsory education	Low
	Diploma	Middle
	Graduation	High
Latvia (I Pudule, unpublished results)^(^ ^46^ ^)^	Elementary	Low
	Secondary	Middle
	Vocational education	
	Higher education	High
Lithuania	No data by maternal education	
Luxembourg† ^(^ ^47^ ^,^ ^48^ ^)^	Primary school education or less	Low
	Professional education	Middle
	Secondary or higher education	High
Malta^(^ ^49^ ^,^ ^50^ ^)^	Primary and secondary education	Low
	Post-secondary and non-tertiary education	Middle
	Tertiary education	High
Netherlands^(^ ^51^ ^,^ ^52^ ^)^	Low	Low
	Middle	Middle
	High	High
Poland (M Kostuch, unpublished results)	Low level of education	Low
	Middle level of education	Middle
	High level of education	High
Portugal	No data by maternal education	
Romania^(^ ^50^ ^,^ ^53^ ^)^	Without primary school	Low
	With primary school	
	Medium studies	Middle
	Superior studies	High
Slovak Republic	No data by maternal education	
Slovenia (M Gabrijelcic, unpublished results)^(^ ^54^ ^)^	Lowest education	Low
	Technical secondary education	Middle
	Academic and professional first cycle degree (Bologna I)	High
	University degree and more	
Spain	No data by maternal education	
Sweden^(^ ^50^ ^,^ ^55^ ^)^	Elementary	Low
	Upper secondary or college≤2 years	Middle
	College ≥3 years or graduate	High
UK: England & Wales^(^ ^50^ ^,^ ^56^ ^)^	Aged 16 years or under when left full-time education	Low
	Aged 17–18 years when left full-time education	Middle
	Aged more than 18 years when left full-time education	High
Norway^(^ ^50^ ^,^ ^57^ ^)^	≤12 years of education	Low
	13–15 years of education	Middle
	≥16 years of education	High
Iceland	No data by maternal education	

ISCED, International Standard Classification of Education.

* Bulgaria: only up to secondary level reported in Bulgarian study making it incomparable with other surveys.

† Luxembourg: secondary and higher education were reported together making it incomparable with other surveys.

Twenty-five nationally representative breast-feeding surveys authorized by national authorities were obtained by year of most recent survey: Austria 2007, Belgium 2013, Croatia 2014, Cyprus 2006, Czech Republic 2009, Denmark 2016, Estonia 2016, Finland 2012 and 2009, France 2016, Germany 2015, Greece 2009, Hungary 2014, Iceland 2012, Ireland 2010, Italy 2014, Latvia 2010, Luxembourg 2010, Malta 2015, Netherlands 2015, Poland 2016, Romania 2011, Slovak Republic 2011, Spain 2016, Sweden 2015 and UK 2012. No national studies were found for five countries and for these only sub-national peer-reviewed studies (Bulgaria 2010, Lithuania 2016, Portugal 2007, Slovenia 2010 and Norway 2010) were found. One peer-reviewed study was found for each of EBF at 4 months in France (2016) and breast-feeding at 1 week in Sweden (2016).

### Maternal education level

Socio-economic position was represented by maternal education level. Education levels were harmonized according to the International Standard Classification of Education (ISCED)^(^ ^23^ ^)^ and defined in three categories: low = levels 0–2 (compulsory, i.e. primary or lower secondary education); middle = levels 3–4 (upper secondary and post-secondary non-tertiary education); and high = levels 5–6 (tertiary education; Table 1).

### Breast-feeding variables

For the purpose of comparison, the following breast-feeding variables used in national surveys were grouped together into one variable called ‘any breast-feeding (ABF)’: breast-feeding within the first hour (recommended by WHO^(^ ^24^ ^)^); ever breast-fed; breast-feeding at all; breast-feeding at birth; and breast-feeding at discharge. In addition, rates on ‘breast-feeding at 1 week’ from Sweden and ‘breast-feeding at <1 month’ from Finland were included in the ABF variable. Three additional ABF variables were created (Table 2) to group the different time frames used in national studies to allow their comparison: ‘ABF at ≤2 months’, which included ABF rates from between 1 and 8 weeks; ‘ABF at ≤4 months’, which included ABF rates from between 8 and 16 weeks; and ‘ABF at 6 months’, which included ABF rates reported at 6 months. Similarly, the wide range of variables used in national surveys to report rates of EBF were grouped together into four variables (Table 3). These were based on the different definitions for time frame used in national studies. The variable ‘EBF at ≤1 month’ included all EBF rates from 1 to 4 weeks. The variable ‘EBF at ≤3 months’ included all EBF rates from 5 to 12 weeks. The variable ‘EBF at 4 months’ included all EBF rates relating to 4 months (e.g. EBF up to 4 months and EBF at least until 4 months). The fourth variable was ‘EBF at 6 months’.

**Table 2:** Any breast-feeding (ABF) rate by level of maternal education in twenty-seven EU countries plus Norway and Iceland, 2006–2016

Country, date of publication (date of data collection)	ABF (%)		ABF at ≤2 months (1–8 weeks) (%)		ABF at ≤4 months (8–16 weeks) (%)		ABF at 6 months (%)	
	Mean	LMH	Mean	LMH	Mean	LMH	Mean	LMH
Austria 2007 (2006)^(^ ^26^ ^)^	93	L = 85			72		55	
		M = 95						
		H = 98						
Belgium 2013 (2012)^(^ ^58^ ^)^	75							
Bulgaria* 2010 (2007)^(^ ^29^ ^)^	95	L = 91	93					
		M = 92*						
Croatia 2014 (2011)^(^ ^59^ ^)^	93							
Croatia 2013 (2012)^(^ ^30^ ^)^		L = 87						
		M = 93						
		H = 95						
Cyprus 2006 (2004)^(^ ^60^ ^)^	79							
Czech Republic 2009 (2007)^(^ ^61^ ^)^	96							
Denmark 2016 (2014)^(^ ^32^ ^)^			90					
Estonia 2016 (2015)^(^ ^62^ ^)^			88					
Finland 2012 (2010–2011)^(^ ^36^ ^)^				L = 85				
				M = 90				
				H = 98				
Finland 2009 (1996–2004)^(^ ^63^ ^)^	99		92				58	
France 2016 (2013)^(^ ^38^ ^)^	66	L = 55						
		M = 60						
		H = 71						
Germany 2015 (2009–2012)^(^ ^39^ ^)^	82	L = 68						
		M = 85						
		H = 95						
Greece 2009 (2006–2008)^(^ ^40^ ^)^	88		54		39	L = 33	22	L = 20
						M = 38		M = 23
						H = 41		H = 24
Hungary 2014 (2012)^(^ ^64^ ^)^	96							
Ireland 2010 (2008–2009)^(^ ^43^ ^)^	57	L = 29						
		M = 54						
	H = 79							
Italy 2014 (2013)^(^ ^45^ ^)^	86	L = 82			42			
		M = 86						
		H = 89						
Latvia 2010 (2010)^(^ ^65^ ^)^	88							
Lithuania 2016 (2015)^(^ ^66^ ^)^					64		45	
Luxembourg† 2010 (2008)^(^ ^47^ ^)^	90	L = 88			45		41	
		M = 87						
		H = 93†						
Malta 2015 (2010 & 2012)^(^ ^49^ ^)^	71	L = 44					38	
		M = 63						
		H = 74						
Netherlands 2015 (2015)^(^ ^52^ ^)^	80	L = 68			59		52	
		M = 81						
		H = 89						
Poland 2016 (M Kostuch, unpublished results)		L = 95						L = 38
		M = 95						M = 47
		H = 95						H = 45
Poland 2014 (2013)^(^ ^67^ ^)^	98		46					
Portugal 2007 (2003)^(^ ^68^ ^)^	91				55		34	
Romania 2011 (2010)^(^ ^53^ ^)^	93				71		49	
Slovenia‡ 2010^(^ ^65^ ^)^	97							
Spain 2016 (2011–12)^(^ ^69^ ^)^			66		54		29	
Sweden 2016 (2004–2010)^(^ ^55^ ^)^				L = 95			63	L = 49
				M = 96				M = 57
				H = 99				H = 79
Sweden 2015 (2013)^(^ ^70^ ^)^	96		86					
UK: England & Wales 2012 (2010)^(^ ^56^ ^)^	81	L = 63	55				34	
		M = 75						
		H = 91						
Norway 2010 (1999–2008)^(^ ^57^ ^)^	99	L = 98	94				80	
		M = 99						
		H = 99						
Iceland 2012 (2004–2008)^(^ ^71^ ^)^	98		92				74	

L, low level of education (ISCED 0–2); M, medium level of education (ISCED 3–4); H, high level of education (ISCED 5–6); ISCED, International Standard Classification of Education.

* Bulgaria: maternal education levels incomparable with other surveys.

† Luxembourg: maternal education levels incomparable with other surveys.

‡ Mean duration of ABF: L = 7 months, M = 9 months, H = 11 months.

**Table 3:** Exclusive breast-feeding (EBF) rate by level of maternal education in twenty-three EU countries plus Norway and Iceland, 2006–2016

Country, date of publication (date of data collection)	EBF at ≤1 month (1–4 weeks) (%)		EBF at ≤3 months (5–12 weeks) (%)		EBF at 4 months (%)		EBF at 6 months (%)	
	Mean	LMH	Mean	LMH	Mean	LMH	Mean	LMH
Austria 2007 (2006)^(^ ^26^ ^)^			60				10	
Belgium 2013 (2012)^(^ ^58^ ^)^			28				8	
Croatia 2014 (2011)^(^ ^59^ ^)^			76				15	
Croatia 2013 (2012)^(^ ^30^ ^)^					38	L = 25		
						M = 32		
						H = 38		
Cyprus 2006 (2004)^(^ ^60^ ^)^			52		15		12	
Czech Republic 2011 (2009)^(^ ^72^ ^)^			35				18	
Denmark 2016 (2014)^(^ ^32^ ^)^	79		69		61	L = 39	17	
						M = 54		
						H = 71		
Estonia 2016 (2015)^(^ ^62^ ^)^	80		64				30	
Finland 2012 (2010–2011)^*^ ^(^ ^36^ ^)^					23		6	
Finland 2009 (1996–2004)^(^ ^63^ ^)^	56		32					
France 2016 (2013)^(^ ^38^ ^)^					30			
France 2013 (2003–2006)^(^ ^73^ ^)^			40			L = 16	18	
						M = 20		
						H = 22		
Germany 2015 (2009–2012)^(^ ^39^ ^)^					34	L = 20		
						M = 35		
						H = 50		
Greece 2009 (2006–2008)^(^ ^40^ ^)^	21	L = 17	10		8		0·5	
		M = 15						
		H = 25						
Hungary 2014 (2012)^(^ ^64^ ^)^					62			
Hungary 2009 (2007)^(^ ^61^ ^)^			95				44	
Italy 2014 (2013)^(^ ^45^ ^)^	49		44		39		6	
Latvia 2016 (2006) (I Pudule, unpublished results)	92		63		53		34	L = 12
								M = 24
								H = 38
Lithuania 2016 (2015)^(^ ^66^ ^)^							31	
Luxembourg† 2010 (2008)^(^ ^47^ ^)^					26†	L = 18	6	L = 6
						M = 16		M = 6
						H = 32		H = 7†
Netherlands 2015 (2015)^(^ ^52^ ^)^	57	L = 50	47	L = 42	45	L = 40	39	
		M = 58		M = 46		M = 45		
		H = 65		H = 54		H = 52		
Poland 2014 (2013)^(^ ^67^ ^)^					29		9	L = 6
								M = 10
								H = 10
Portugal 2007 (2003)^(^ ^68^ ^)^			40				17	
Romania 2011 (2010)^(^ ^53^ ^)^	56		50		25		13	L = 14
								M = 12
								H = 15
Slovak Republic 2011 (2010)^(^ ^74^ ^)^	90		73		64		49	
Sweden 2015 (2013)^(^ ^70^ ^)^	83		67		53		15	
UK: England & Wales 2012 (2010)^(^ ^56^ ^)^	30		17		12		1	
Norway 2010 (1999–2008)^(^ ^57^ ^)^	85		71		44		2	
Iceland 2012 (2004–2008)^(^ ^71^ ^)^	86		67		50		8	

L, low level of education (ISCED 0–2); M, medium level of education (ISCED 3–4); H, high level of education (ISCED 5–6); ISCED, International Standard Classification of Education.

* Median duration of EBF: L = 1 month, M = 1·4 months, H = 2 months.

† Luxembourg: maternal education levels incomparable with other surveys.

### Inequality gap

The inequality gap analysis was carried out by calculating the difference between the breast-feeding rates of mothers with the highest minus the lowest level of education, and expressed as a percentage of the rate in the highest education level^(^ ^25^ ^)^. The breast-feeding rates of mothers with the highest level of education were used as the highest possible achievable rate for that specific country when calculating the inequality gaps.

### Statistical analysis

All data were inserted and cleaned in MS Excel (Microsoft Office 2016). Relative breast-feeding rates with reference to high level of maternal education were computed for middle and low levels of maternal education. For statistical testing, breast-feeding percentage values in the groups by level of maternal education were converted to arcsine values to apply parametrical testing. For comparisons between multiple groups, ANOVA with the *post hoc* Tukey honest significant difference test was applied and *P* <0·05 was considered to indicate statistical significance. Breast-feeding cessation rates were evaluated with the matched-pair *t* test. The statistical software package IBM SPSS Statistics version 24 was used for statistical testing.

## Results

The levels of maternal education reported are listed in Table 1 ^(^ ^26^ ^–^ ^57^ ^)^. No breast-feeding rates by level of education were found for one-third (*n* 10) of the countries (Belgium, Cyprus, Czech Republic, Estonia, Hungary, Lithuania, Portugal, Slovak Republic, Spain and Iceland). The levels of mothers’ education were grouped into high, medium or low categories using the ISCED classification for those twenty countries that did report breast-feeding rates by level of education. Only eighteen countries could be compared based on the ISCED classification since education levels in Bulgaria and Luxembourg could not be matched to the ISCED classification. Bulgaria’s and Luxembourg’s highest level of education included ‘secondary’ or ‘secondary or higher’, respectively, whereas the ISCED levels 5–6 (i.e. high) include only tertiary education plus bachelor or equivalent and higher but not secondary.

### Any breast-feeding

Data on average rates of ABF were found for all except one country (Slovak Republic; Table 2 ^(^ ^26^ ^,^ ^29^ ^,^ ^30^ ^,^ ^32^ ^,^ ^36^ ^,^ ^38^ ^–^ ^40^ ^,^ ^43^ ^,^ ^45^ ^,^ ^47^ ^,^ ^49^ ^,^ ^52^ ^,^ ^53^ ^,^ ^55^ ^–^ ^71^ ^)^). The dates when ABF rates were published ranged from 2006 to 2016: the oldest in Cyprus in 2006; Austria and Portugal in 2007; Greece in 2009; six countries (Bulgaria, Latvia, Ireland, Luxembourg, Slovenia and Norway) in 2010; three countries (Czech Republic, Romania and Slovak Republic) in 2011; three countries (Finland, UK and Iceland) in 2012; Belgium in 2013; three countries (Croatia, Hungary and Italy) in 2014; three countries (Germany, Malta and Netherlands) in 2015; and seven countries (Denmark, Estonia, France, Lithuania, Poland, Spain and Sweden) in 2016. In addition to the wide time span between the dates of publication, each country also used different time frames and variables for reporting their ABF rates. The time frames ranged from infant ever breast-fed, initiation at birth, within the first hour, at discharge and from breast-feeding at a range of weeks and/or months, up to 6 months. This resulted in about seventy different variables for ABF. In an attempt to compare the wide range of time frames they were grouped into four: any breast-feeding (ABF); ABF at ≤2 months (1–8 weeks); ABF at ≤4 months (8–16 weeks); and ABF at 6 months (Table 2).

Average ABF rates above 90 % were found in thirteen countries: Finland (99 %), Norway (99 %), Iceland (98 %) Poland (98 %), Slovenia (97 %), Czech Republic (96 %), Hungary (96 %), Sweden (96 %), Bulgaria (95 %), Austria (93 %), Croatia (93 %), Romania (93 %) and Portugal (91 %; Table 2). Average ABF rates between 80 and 90 % were found in seven countries: Luxembourg (90 %), Greece and Latvia (88 %), Italy (86 %), Germany (82 %), UK (81 %) and Netherlands (80 %). Average ABF rates between 70 and 80 % were found in three countries: Cyprus (79 %), Belgium (75 %) and Malta (71 %). The lowest rates of ABF were found in France and Ireland, 66 and 57 %, respectively. Average ABF rates at ≤2 months were highest (>80 %) in Nordic countries, Estonia and Bulgaria: Norway (94 %), Bulgaria (93 %), Iceland (92 %), Finland (92 %), Denmark (90 %), Estonia (88 %) and Sweden (86 %). Average ABF rates above 50 % at 4 months were found in six countries: Austria (72 %), Romania (71 %), Lithuania (64 %), Netherlands (59 %), Portugal (55 %) and Spain (54 %); and average ABF rates below 50 % at 4 months in Poland (46 %), Greece (39 %), Luxembourg (45 %) and Italy (42 %). Average ABF rates at 6 months were reported by fourteen countries, where only Nordic countries, Austria and the Netherlands reported rates above 50 %: Norway (80 %), Iceland (74 %), Sweden (63 %), Finland (58 %), Austria (55 %) and Netherlands (52 %). The lowest ABF rates at 6 months were reported in the UK and Mediterranean countries: Malta (38 %), UK (34 %), Portugal (34 %), Spain (29 %) and Greece (22 %).

### Any breast-feeding disparities

**Fig. 1:**
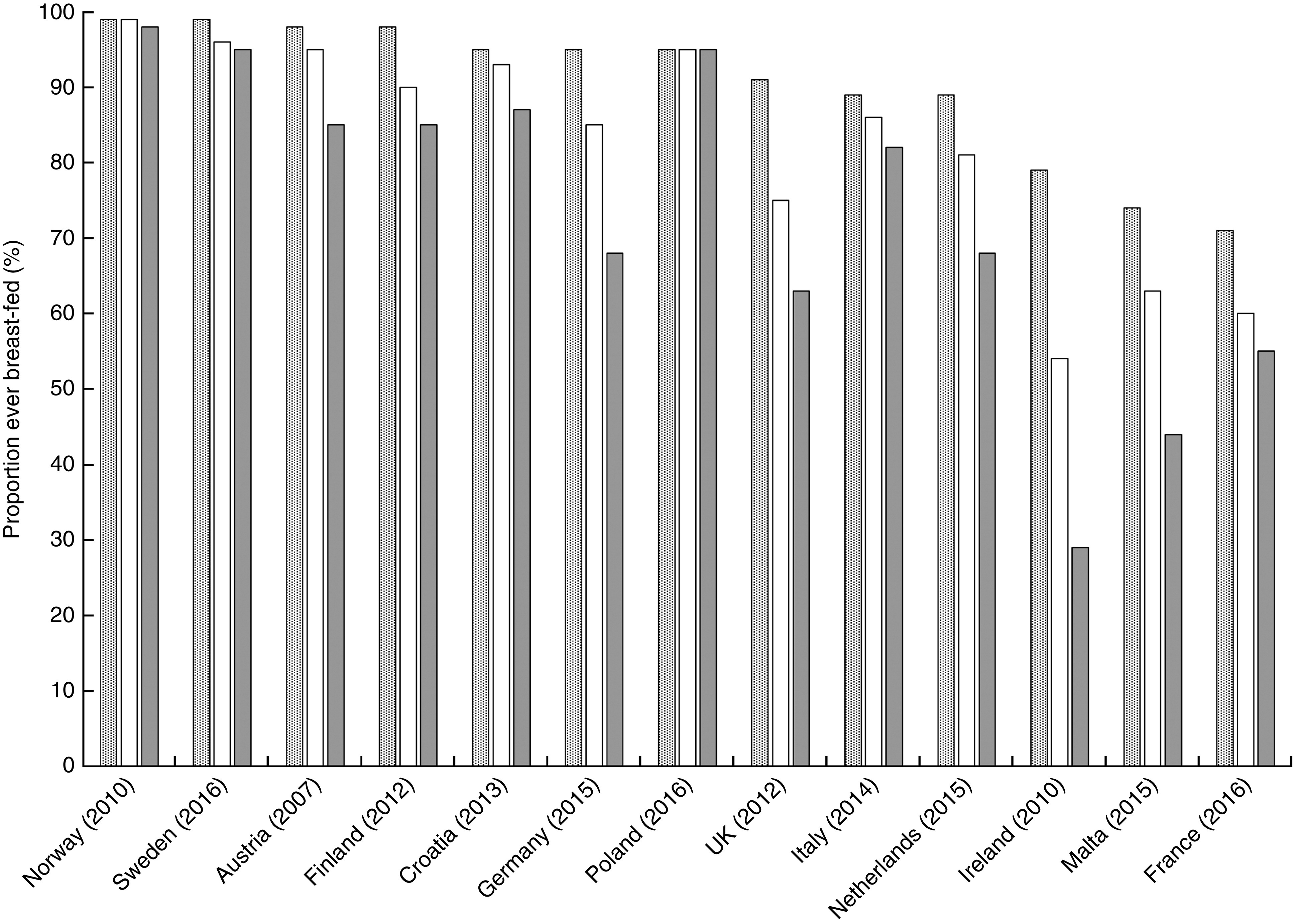
Any breast-feeding rate according to high (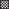), middle (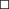) and low (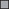) maternal education levels in twelve EU countries plus Norway, 2006–2016

**Fig. 2:**
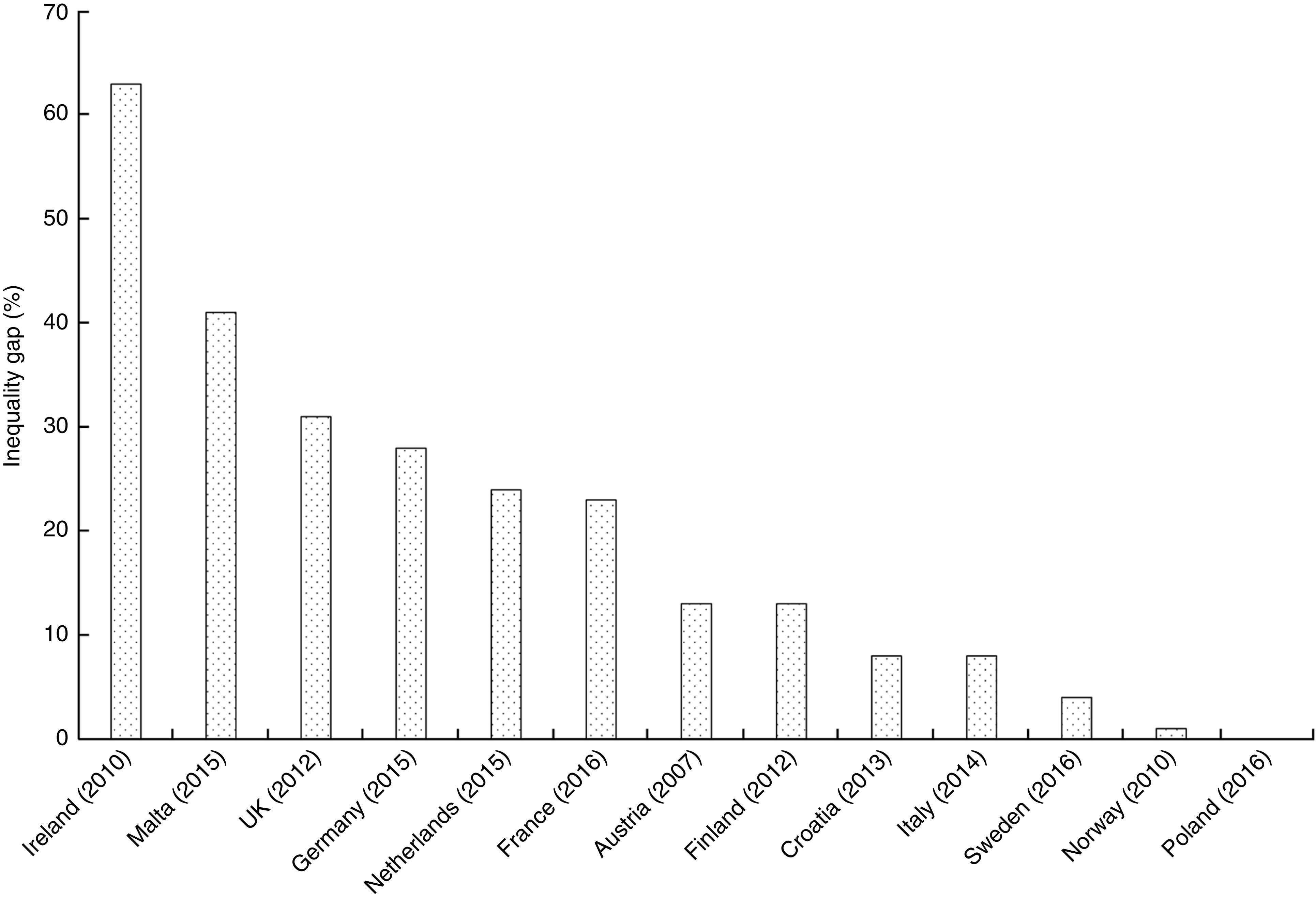
Inequality gap in any breast-feeding rate between maternal high compared with low education level in twelve EU countries plus Norway, 2006–2016. Inequality gap (%) = [(proportion of breast-feeding mothers with high level of education – proportion of breast-feeding mothers with low level of education)/proportion of breast-feeding mothers with high level of education] × 100

Fifteen countries reported rates of ABF disaggregated by education level but the data from Bulgaria and Luxembourg could not be compared with the others. Among the thirteen countries that were compared, there is a significant difference in ABF rates between mothers with high, medium and low education levels. The proportion of mothers with high education levels, practising ABF, is on average 20 % higher (*P* = 0·035) than those with low levels. Figure 1 illustrates a clear tendency towards a social gradient in breast-feeding rates between the mothers according to high, middle and low level of education. However, some countries have less steep social gradients as shown by the very small percentage inequality gaps in the Polish, Norwegian and Swedish mothers with high and low education levels (Fig. 2). In Poland, Norway and Sweden, more than 90 % of mothers are breast-feeding regardless of their level of education and so the inequality gaps are minor. Small disparities are observed in Italy and Croatia (<10 %), with relatively larger inequality gaps in Finland and Austria (<20 %) and in France, the Netherlands and Germany (<30 %). The largest inequality gaps are observed in the UK (31 %), Malta (41 %) and Ireland (63 %; Fig. 2). In five countries (Greece, Poland, Sweden, Luxembourg and Netherlands), where rates are reported at more than one time point during the first 6 months, there is a marked rapid cessation after initiation. The cessation gradient tends to be steeper in mothers with low level of education as they stop breast-feeding earlier (mean 71 (sd 17) %), compared with those having high education level (mean 62 (sd 17) %; *P* = 0·036). Similarly, at 6 months, the inequality gap in ABF of Swedish mothers increases to 38 % as more mothers with low education level stop breast-feeding earlier compared with those with higher education. Low levels of maternal education are not only associated with low rates of initiation but also with a higher probability of early cessation.

### Exclusive breast-feeding at 6 and 4 months

Only six countries (Bulgaria, Germany, Ireland, Malta, Slovenia and Spain) did not report the average EBF rates at 6 months (Table 3 ^(^ ^26^ ^,^ ^30^ ^,^ ^32^ ^,^ ^36^ ^,^ ^38^ ^–^ ^40^ ^,^ ^45^ ^,^ ^47^ ^,^ ^52^ ^,^ ^53^ ^,^ ^56^ ^–^ ^64^ ^,^ ^66^ ^–^ ^68^ ^,^ ^70^ ^–^ ^74^ ^)^). At 6 months, the highest average EBF rates were reported in Slovakia (49 %) and Hungary (44 %) and the lowest in the UK (1 %) and Greece (0·5 %; Table 3). The WHO recommendation to Member States used to be to collect national data on EBF rates at 4 months, but in the year 2000 this recommendation was increased to 6 months. However, many countries continue to collect data on EBF rates at 4 months (Table 3). The highest average rates of EBF at 4 months (≥50 %) were observed in six countries: Slovak Republic, Hungary and Denmark (64, 62 and 61 %, respectively) followed by Sweden, Latvia and Iceland (53, 53 and 50 %, respectively). The lowest average EBF rates at 4 months (<20 %) were in Cyprus, the UK and Greece (15, 12 and 8 %, respectively).

### Exclusive breast-feeding disparities at 6 and 4 months

Only four countries, Latvia, Luxembourg, Poland and Romania, reported rates of EBF at 6 months by level of maternal education (Table 3). In Poland and Latvia, two to three times more mothers with high compared with low education level practised EBF at 6 months. In both Romania and Luxembourg there was little difference in EBF rates at 6 months between mothers with high compared with low education level (Table 3). Six countries reported EBF rates at 4 months by level of maternal education (Table 3): more Danish and German mothers with high compared with low education level practised EBF and the inequality gaps were 45 and 60 %, respectively. Similarly, a greater proportion of Dutch, French, Croatian and Luxembourg mothers with high compared with low education level practised EBF at 4 months and the respective inequality gaps were 23, 27, 34 and 44 % at 4 months.

Finally, Table 4 lists the wide range of different time frames used to define breast-feeding rates, for example: initiation of breast-feeding at birth; initiation of breast-feeding within the first hour; any breast-feeding; ever breast-feeding; breast-feeding at discharge from hospital; breast-feeding at 1 week; and breast-feeding at or less than 1, 2, 3 and 4 months. This wide range of non-standardized time frames makes comparison between countries impractical.

**Table 4:** Different breast-feeding time frames used within the thirty European countries

Time frame	Number of countries	Disaggregated by maternal education	Breast-feeding rate (%), minimum–maximum
Infant ever breast-fed	25	13	57–99
Breastfeeding within the first hour or at birth	6	0	5–93
Breast-feeding at discharge	3	0	58–88
ABF at <2 months (from 1 up to 8 weeks)	11	2	46–94
ABF at ≤4 months (from 9 up to 16 weeks)	9	1	39–72
ABF at 6 months	14	3	22–80
ABF at 1 year	8	0	8–27
EBF at ≤1 month (from 1 up to 4 weeks)	13	2	21–92
EBF at ≤3 months (from 5 up to 12 weeks)	21	1	10–95
EBF at/up to/until 4 months (from 12 up to 16 weeks)	19	6	8–64
EBF at/up to/until 6 months^*^ (from 16 up to 24 weeks/6 months)	24	4	0·5–49

ABF, any breast-feeding; EBF, exclusive breast-feeding.

* WHO recommendations changed from 4 months of EBF to 6 months of EBF in 2000.

## Discussion

The current paper presents information comparing the rates of breast-feeding in Europe in mothers of different SES, defined by level of maternal education. The results confirm what other authors have stressed concerning the need for standardized methods to monitor breast-feeding rates in Europe to allow comparison between countries and recommendations to reduce inequalities^(^ ^75^ ^,^ ^76^ ^)^.

### Any breast-feeding and disparities

The results confirm previous studies and provide new comparative evidence that disparities exist, not only within but also between countries^(^ ^6^ ^,^ ^77^ ^)^. As indicated, based on surveys from thirteen countries, one-fifth more mothers with high compared with low education level breast-fed. Whereas the initial disparities in the Nordic, Baltic and Central European countries are relatively small, the inequality gaps found in Ireland, Malta, the UK, Germany, the Netherlands and France are high and should give cause for concern to the respective governments (Fig. 2). When breast-feeding rates are reported only as national averages, these average figures can conceal a steep gradient between mothers with high compared with low education level. For example McAndrew *et al*.^(^ ^56^ ^)^ found that the incidence of ABF was 91 % among UK mothers with high education level compared with 63 % among those with low education level. Similarly, five countries (Greece, Poland, Sweden, Luxembourg and Netherlands) reported rates over two or three time periods during which the trend is that the inequality gaps get wider. Even Nordic countries, for example Sweden, report that by 6 months the inequality gap increases considerably as more Swedish mothers with low education stop breast-feeding earlier than those with higher education. This is confirmed by Wallby and Hjern^(^ ^78^ ^)^, who found that low income levels are a strong predictor for early cessation of breast-feeding and recommended extra protection for low-income Swedish mothers. Similarly Finnish^(^ ^10^ ^,^ ^79^ ^,^ ^80^ ^)^ and Danish studies^(^ ^81^ ^)^ reported that mothers with low compared with high education were more likely to introduce foods too early. Indeed, even Norwegian authors recently reported that very few (7 %) Norwegian mothers with low education level still exclusively breast-fed by 5 months compared with three times more mothers (22 %) with high level^(^ ^82^ ^)^.

### Exclusive breast-feeding and disparities

The results confirm that on average the EBF rates in most countries, except the Slovak Republic (49 %) and Hungary (44 %), are well below governments’ 2025 target of at least 50 % EBF at 6 months^(^ ^1^ ^)^. Victora *et al*.^(^ ^2^ ^)^ confirm that Nordic countries have high breast-feeding initiation rates of over 90 %. Similarly, Baltic countries and those in Central Europe report high initiation rates, with over 90 % in Bulgaria, Croatia, Czech Republic, Hungary, Poland, Slovak Republic and Slovenia. Even at 6 months about one-third of Baltic (Latvian, Lithuanian and Estonian) mothers are still practising EBF (Table 3). Results from smaller studies^(^ ^83^ ^,^ ^84^ ^)^ report that, on average, about one-half of Lithuanian mothers exclusively breast-feed for 6 months, but only one-fifth with low compared with four-fifths of those with high level of education continue until 6 months. Slovakia and Hungary, along with the Baltic and Nordic countries, report the highest EBF rates (Table 3) and interestingly these countries provide the best statutory entitlement of paid parental leave, closely followed by Czech Republic and Bulgaria^(^ ^85^ ^)^. In contrast, French mothers can only expect 6 weeks off work before and 10 weeks after birth, and even those with high education level have one of the lowest breast-feeding initiation rates (71 %) in Europe (although dramatically increased since 1995)^(^ ^86^ ^,^ ^87^ ^)^. Parental leave payment rates are also low in Ireland and the UK, where fully paid maternity leave lasts only 9 and 12 weeks, respectively^(^ ^85^ ^)^. Furthermore it appears that French, Irish and British mothers are subjected to aggressive marketing and many violations are reported against the International Code of Marketing of Breast Milk Substitutes (‘the Code’ hereafter) in France^(^ ^88^ ^)^, Ireland^(^ ^89^ ^)^ and the UK^(^ ^90^ ^)^. Robust policies, better maternity leave and protection against marketing are needed not only to achieve the global target of at least 50 % EBF in the first 6 months by 2025^(^ ^1^ ^)^ but also to achieve the EU Council’s aim^(^ ^91^ ^)^ to halt the rise in childhood obesity, which is closely associated to poor infant feeding practices. For example, parents have the right to receive information on infant feeding practices, from health professionals, that is independent of commercial interests and free from conflicts of interest. Low levels of breast-feeding have been attributed to lack of knowledge about breast-feeding and incorrect advice on infant feeding from health professionals in Lithuania^(^ ^92^ ^)^, Malta^(^ ^93^ ^)^ and Romania^(^ ^94^ ^)^.

### Implications for public health policy

A recent German study^(^ ^95^ ^)^ is among the first to demonstrate a widening socio-economic disparity in breast-feeding practices using a time-trend analysis. This disparity was largely explained by the proportion of German mothers with low SES who were likely to be obese and/or to smoke. Both these risk factors are well known to be associated with low breast-feeding rates^(^ ^96^ ^,^ ^97^ ^)^. Breast-feeding cessation among Dutch mothers with low education level also increases rapidly^(^ ^98^ ^)^ and so in the Netherlands there is increased focus on how to reduce health inequalities in pregnant women and their offspring^(^ ^99^ ^)^. Similar calls are being made in Italy, where authors stress the need for policies that specifically support mothers with low level of education^(^ ^100^ ^,^ ^101^ ^)^. In the future it is crucial to carry out regular monitoring of trends of breast-feeding rates along with the relative prevalence of obesity. The latest data (2015–2017) from the WHO Childhood Obesity Surveillance Initiative^(^ ^102^ ^)^ show that Southern European countries have the highest rate of child obesity. In Cyprus, Greece, Italy, Malta and Spain, approximately one in five boys is obese, whereas Nordic and Baltic countries are among the countries with the lowest rates. Obesity in young children is associated with low initiation rates of breast-feeding along with early cessation^(^ ^103^ ^,^ ^104^ ^)^. This both negatively affects long-term health and increases the risk of obesity over the next generations^(^ ^105^ ^)^. However, data on both national breast-feeding rates and obesity prevalence, disaggregated by level of maternal education, are needed to better investigate the complex relationship between obesity, breast-feeding and SES. A recent review^(^ ^106^ ^)^ tried to separate independent outcomes but a clearer understanding of the several different mechanisms involved, and their interrelationships, is of utmost importance. The European Blueprint for Action on Breast-feeding^(^ ^75^ ^)^ especially recommends: full implementation of the Code and subsequent relevant World Health Assembly resolutions; maternity protection legislation that enables all working mothers to exclusively breast-feed their infants for 6 months; establishing standards for best practice within public places as well as in all maternity and childcare services; and harmonized collection, at least every 3–5 years, of disaggregated breast-feeding data that correspond to the recommended standardized indicators and definitions.

### Limitations

As these comparisons are derived from data gathered using different methods over different time periods, reported rates and their comparison should be treated with caution^(^ ^107^ ^)^. Breast-feeding rates were collected on different dates over a long time period and a wide range of methods was used in different countries to collect breast-feeding rates (Table 4). It was attempted to harmonize education levels into three categories according to the ISCED^(^ ^108^ ^)^; however, our categorizations could be flawed. Furthermore, reported breast-feeding rates were not always nationally representative and so comparisons should be interpreted with caution. The inequality gap analyses are useful but limited in that they only reflect the difference between low level of maternal education compared with high. The analysis can be potentially affected by extreme values for each of these groups and does not necessarily reflect the experience of the entire population due to the different distribution of mothers’ education levels^(^ ^109^ ^)^.

## Conclusion

Overall, there is less initiation of breast-feeding and shorter duration of EBF by mothers with low compared with high education level. These findings can help governments to investigate why these disparities exist and why some countries such as the Nordic, Baltic and Central European countries have higher breast-feeding rates and lower disparities compared with other European countries. National policies common to Nordic, Baltic and Central European countries are that they have paid maternity leave regulations which go beyond the minimum stipulated by EU law. In addition, Nordic countries have been efficient in minimizing violations of the Code and until recently Baltic and Central European countries have not been as exposed to high levels of marketing by companies selling breast milk substitutes. The results highlight disparities in breast-feeding which can only be reduced if governments develop harmonized monitoring systems that are carried out regularly and where the data are disaggregated by SES to develop appropriate health promotion policies.
